# Lichen Planus Pigmentosus with True Melanocytic Nests: A Case Report with a Comprehensive Literature Review

**DOI:** 10.3390/dermatopathology10030036

**Published:** 2023-09-08

**Authors:** Antonio Podo Brunetti, Gianmarco Diego Bigotto, Giorgio Stabile, Valentina Caputo, Lucia Brambilla, Stefania Guida, Franco Rongioletti

**Affiliations:** 1 Unit of Clinic Dermatology, Vita-Salute San Raffaele University, 20132 Milan, Italy; 2 Department of Surgical Pathology, ASST Grande Ospedale Metropolitano Niguarda, 20162 Milan, Italy; 3 Unit of Dermatology, Foundation IRCCS Ca’ Granda Ospedale Maggiore Policlinico, 20122 Milan, Italy; 4 Unit of Clinic Dermatology, IRCCS San Raffaele Scientific Institute, 20132 Milan, Italy

**Keywords:** lichen planus pigmentosus, lichenoid dermatoses, melanocytic nests, pseudomelanocytic nests, atypical melanocytic proliferation

## Abstract

Lichen Planus Pigmentosus (LPP) is an uncommon variant of lichen planus characterized by the development of dark greyish-brown macules and patches primarily affecting sun-exposed areas. Histologically, it presents with lichenoid interface dermatitis with many melanophages. In select cases, the presence of melanocytic nests or pseudomelanocytic nests within LPP lesions has been documented, posing a diagnostic challenge. We present a detailed case report of a 32-year-old Eritrean woman with a longstanding history of hyperpigmented macules, alongside an in-depth review of the existing literature on lichenoid dermatoses featuring melanocytic or pseudomelanocytic nests. This paper delves into the clinical presentation, histopathological features, differential diagnosis, and potential mechanisms underlying this intriguing phenomenon.

## 1. Introduction

Lichen Planus Pigmentosus (LPP) is a distinctive subtype of lichen planus that predominantly impacts individuals with darker pigmented skin. Lichenoid interface dermatitis with many melanophages is the typical histopathological presentation [1]. The microscopic identification of pseudomelanocytic nests or true melanocytic nests within LPP lesions introduces complexity to the diagnostic process and stimulates inquiries into the intricate interplay between inflammatory processes and melanocyte biology. 

## 2. Case Report

**Figure 1:**
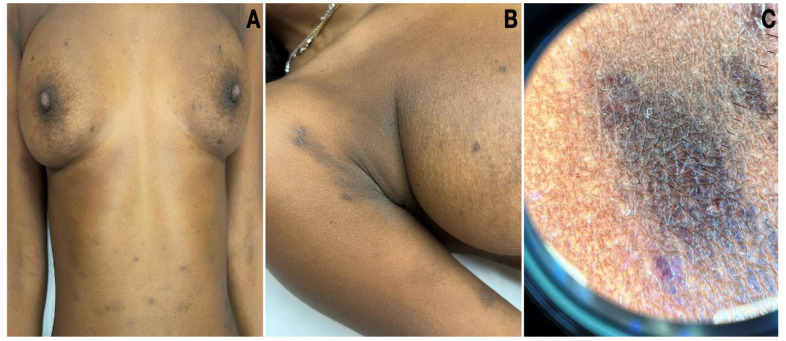
Clinical pictures of LPP. (**A**) Oval grey-brown patches and macules located on the trunk and upper extremities. (**B**) Hyperpigmentation involving the flexure. (**C**) Dermatoscopy of a hyperpigmented patch on the abdomen revealed a diffuse, blotchy pattern.

A 32-year-old Eritrean woman exhibited hyperpigmented macules that manifested between the ages of eight and ten. Initially, small, grey-brown macules emerged mainly on sun-exposed areas and were, at times, associated with mild pruritus. The condition progressively worsened after the age of 30, extensively involving her limbs and trunk. Notably, she had a history of autoimmune thyroiditis, and her familial background featured similar skin lesions on her father and brother. On examination, oval greyish-brown patches and macules located on the trunk, axillary flexures, and upper and lower extremities were seen (Figure 1A,B). The oral mucosa and nails were not involved. The dermatoscopy of a patch on the abdomen revealed a diffuse, blotchy pattern (Figure 1C).

**Figure 2:**
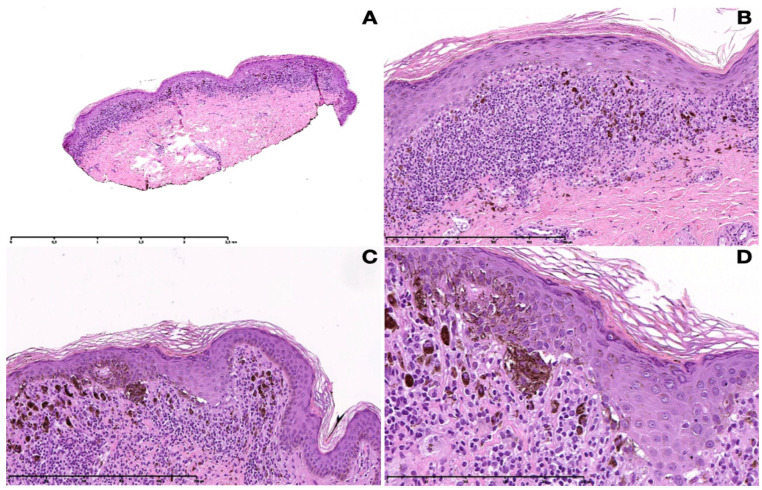
Histopathological pictures of LPP. (**A**) Lichenoid band-like lymphohistiocytic inflammatory infiltrate with numerous melanophages within the papillary dermis (10×). (**B**) Acanthosis with flattening in places, orthohyperkeratosis, and hypergranulosis. Vacuolar degeneration of the basal cell layer with keratinocyte apoptosis and a band-like lymphohistiocytic infiltrate with numerous melanophages (100×). (**C**) Nesting of melanocytes at the dermo-epidermal junction (100×). (**D**) Close-up of junctional nests (200×).

The routine laboratory data were within normal limits or negative. The histopathological examination of a hyperpigmented lesion on the hip showed a band-like lymphohistiocytic inflammatory infiltrate with numerous melanophages within the papillary dermis (Figure 2A,B). Additionally, the vacuolar degeneration of the basal cell layer with keratinocyte apoptosis was evident. The overlying epidermis showed irregular acanthosis with flattening in places, orthohyperkeratosis, and hypergranulosis. Most remarkably, junctional aggregates of melanocytes were present at the dermo-epidermal junction (Figure 2C,D).

**Figure 3:**
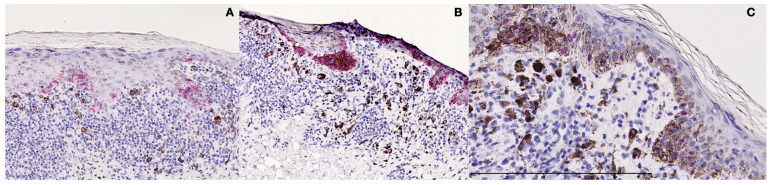
Immunohistochemical studies. (**A**) The cells in the junctional aggregates were found to be HMB-45 positive. (**B**) The cells in the junctional aggregates were found to be Melan-A positive. (**C**) The cells in the junctional aggregates were found to be SOX-10-positive, confirming that the aggregates were “true melanocytic nests”.

Immunohistochemically, the cells in the junctional aggregates were found to be Melan-A-, S-100-, HMB-45-, and SOX-10-positive, confirming that the aggregates were “true melanocytic nests” (Figure 3A–C); CD68 and CD3 were considered negative in the same aggregates.

The differential diagnosis included lichenoid dermatitis versus atypical melanocytic proliferation. Ultimately, a conclusive diagnosis of LPP with true melanocytic nests was established based upon the clinico-pathological correlation. Therapy with acitretin associated with extreme sun protection was started with a slight improvement after 6 weeks.

## 3. Discussion

LPP predominantly emerges in adulthood, typically manifesting insidiously after the age of 30. Small black or greyish-brown macules on sun-exposed areas tend to evolve into larger hyperpigmented patches. While the face, trunk, flexures, and upper extremities are commonly affected, the oral mucosa, palms, soles, and nails remain unaffected. The precise etiology of LPP remains elusive; however, the potential triggers include sunlight exposure, hepatitis C virus, mustard oil, and cosmetic agents. Abnormalities in T-lymphocyte functions have been implicated, and an intriguing association with thyroid disorders, such as in our case, has been noted [2].

Our case is peculiar for the discovery of junctional “true melanocytic nests” on histopathologic grounds. The phenomenon of junctional nesting or pseudonesting in the setting of microscopic lichenoid/interface changes, although infrequently and regardless of the terms used, has been previously reported [3,4,5,6,7,8,9]. The current state of the art seem to categorize this rare phenomenon into three different microscopic groups: “pseudomelanocytic nests” that are composed of macrophages, keratinocytes, and melanocytic debris without melanocytes; “melanocytic pseudonests” or “mixed nests” that are composed of both keratinocytes, inflammatory cells, and melanocytes; and “true melanocytic nests” made exclusively of melanocytes. The phenomena of junctional nesting and pseudonesting have been observed in various interface lichenoid dermatoses. Thirty-one cases have been identified in the literature, including ours, with a prevalence of 20 males (Table 1) [3,4,5,6,7,8,9,10,11,12,13,14,15,16]. The main diagnoses were cutaneous LE in six cases, lichen planus in four cases, fixed drug eruption in four cases, and a range of conditions with a lichenoid pattern including LPP and lichenoid keratoses, and even graft-vs.-host-disease (Table 1). Most of the cases exhibited a “pseudomelanocytic nests” pattern on histopathological grounds, while the presence of “true melanocytic nests” confirmed by the positivity of nuclear panmelanocytic markers, such as SOX-10 or MITF-1, were found in only a few cases, including ours. Specifically, four cases of LPP have been previously described, but all the patients presented with a “pseudomelanocytic nest” or “melanocytic pseudonest” pattern [6,10,12], which makes them different from our case that seems to be the only one with a “true melanocytic” pattern. However, most provided studies in Table 1 consistently reveal the positive reactivity of these nests to at least one melanocytic marker, often with multiple markers. It is noteworthy that the studies wherein nests exhibited negativity with melanocytic markers pertain to the oral mucosa, potentially lacking relevance to the cutaneous manifestation of LPP.

As for pathogenesis, the development of true melanocytic nests within LPP lesions may stem from a reorganization of the “epidermo-melanocytic” unit, which is triggered by inflammatory changes in the dermo-epidermal junction. The disruption of the basement membrane zone may activate cytokines or signaling pathways that contribute to the recruitment and proliferation of residual epidermal melanocytes. The identification of true melanocytic nests in LPP requires differentiation from atypical junctional melanocytic proliferations, especially in cases involving sun-damaged skin. In our case, the absence of pagetoid spreading, the lack of confluence of single melanocytes at the dermo-epidermal junction, and significant cytologic atypia associated with typical lichen planus-like features on histopathological grounds along with the clinical features were not consistent with an atypical melanocytic proliferation and favored a diagnosis of LPP with “true melanocytic nests” [6]. Clinicopathological correlation plays a pivotal role in achieving an accurate diagnosis. The collision of lichen planus with a melanocytic nevus (pseudomelanoma-like changes) in conditions like lichen sclerosus, epidermolysis bullosa (“GABEB nevus”), and post-inflammatory states following toxic epidermal necrolysis, burns, and other inflammatory disorders affecting the junctional area of melanocytic nevi further highlights the complexity of histopathological differential diagnosis that can be easily resolved via clinicopathological correlation.

Our patient reported that some identical hyperpigmented lesions were present in her father and brother, but unfortunately, the histopathological analysis of her relatives is not possible for confirmation. Consequently, we are unable to definitively ascertain the potential occurrence of the familial manifestation of LPP, an exceedingly rare phenomenon. While the influence of genetic factors remains uncertain, instances of Lichen Planus (LP) affecting multiple family members have been reported. Notably, a case involving two monozygotic twin sisters with oral LP has been documented, and one of them additionally exhibited LPP [17].

Therapy is difficult and consists of the avoidance of triggers and topical and systemic medications to stop the inflammatory reaction and reduce pigmentation. Drugs including potent topical corticosteroids, oral steroids, topical calcineurin inhibitors, antimalarials, topical and systemic retinoids, and skin-lightening agents were recommended for LPP [2].

## 4. Conclusions

The presence of true melanocytic nests in LPP lesions highlights the intricate nature of cutaneous inflammatory disorders and their potential to mimic melanocytic proliferations. Accurate diagnosis necessitates rigorous clinicopathological correlation and heightened awareness of this infrequent phenomenon among dermatologists and dermatopathologists. A multifaceted understanding of LPP, underscored by the occurrence of true melanocytic nests, contributes to a more nuanced comprehension of the complex interplay between inflammation and melanocyte behavior.

## Figures and Tables

**Table 1:** Review of all cases of melanocytic nests in lichenoid dermatitis [3,4,5,6,7,8,9,10,11,12,13,14,15,16].

Reference	Age/Sex	Clinical Presentation and Location	Positive/Negative Nests Stains	Final Diagnosis	Interpretation of Nests
Maize et al., 2003 [3]	35/M	Blue-gray macules on the left temple	Melan-A + HMB45, S100, Tyr, BCL2−	Discoid and melanotic LE	Pseudomelanocytic nests
Beltraminelli et al., 2009 [10]	60/M	Ill-defined brown grayish pigmentation on the checks	Melan-A, CK + HMB45, S100−	Lichenoid phototoxic reaction	Pseudomelanocytic nests
Beltraminelli et al., 2009 [10]	59/M	Irregular, partly confluent, reticulated pigmentation on the forehead	Melan-A, CK + HMB45, S100−	LPP	Pseudomelanocytic nests
Beltraminelli et al., 2009 [10]	52/F	Small scaly plaque of the infraorbital region	Melan-A, CK + HMB45, S100−	Pigmented lichenoid keratosis	Pseudomelanocytic nests
Nicholson and Gerami 2010 [11]	39/F	New asymptomatic brown macules along the hair line	Melan-A, CK, CD68, CD3 + HMB45, S100, MITF−	FDE	Pseudomelanocytic nests
Nicholson and Gerami 2010 [11]	76/F	Well-demarcated hyperpigmented patch on the periocular arca	Melan-A, CK, CD3 + HMB45, S100, MITF, CD68 −	FDE	Pseudomelanocytic nests
Silva et al., 2011 [12]	48/M	Discrete, reticulate, hyperpigmented patch of the neck	Melan-A, S100, SOX10, MITF, CK + −	LPP/Erythema dyschromicum perstans	Melanocytic pseudonests
Boros et al., 2014 [13]	66/M	Solitary slightly elevated pigmented lesion on the interdental papilla	CD68 + Melan-A, SOX10, MITF−	Chronic mucositis	Pseudomelanocytic nests
Boros et al., 2014 [13]	61/F	Solitary pigmented lesion on the hard plate	CD68 + Melan-A−	Melanophages	Pseudomelanocytic nests
Chung et al., 2015 [14]	40/M	Violaceous, polygonal, flat-topped, mildly pruritic papules sited on the back, the ankles, and feet	Melan-A, SOX10+ −	LP	Melanocytic nests
Hall et al., 2015 [15]	48/M	Irregular brown and violaceous patch on the chest	Melan-A, SOX10, MITF, S100 + −	LP actinicus	Melanocytic pseudonests
Pantaleão et al., 2016 [16]	47/F	Pigmented lesion of the left upper arm	Melan-A, HMB45, S100, TYR + −	FDE	Pseudomelanocytic nest
Pantaleão et al., 2016 [16]	34/M	Greyish brown plaque, scattered black papules, and greyish macules on the left forehead and right side of the face.	Melan-A + −	Vacuolar interface dermatitis	Pseudomelanocytic nests
Venturini et al., 2018 [4]	40/M	Asymptomatic pigmented patch on the right cheek	Melan-A + −	Pigmented actinic LP	Melanocytic pseudonests
Dos Santos et al., 2018 [5]	41/F	Irregularly pigmented lesion between the left maxillary central and lateral incisors	Melan-A, CK, CD68 + SOX10, S100−	Oral melanocanthoma	Pseudomelanocytic nests
McClanahan D et al., 2018 [6]	32/M	Speckled brown appearance and areas of erythema on the anterior shoulder	Melan-A, S100, CK + SOX10−	Lichen striatus	Melanocytic pseudonests
Ferrara et al., 2020 [7]	36/M	Brownish macule on the left temple	Melan-A, SOX10, MITF + S100, CK, PRAME−	Melanotic cutaneous LE	Melanocytic nests
Ferrara et al., 2020 [7]	31/M	Linear atrophic hyperpigmented patch on the neck and the chin	Melan-A, SOX10, MITF, CK + S100, PRAME−	Discoid LE	Melanocytic pseudonests
Ferrara et al., 2020 [7]	66/F	Bilateral symmetric grayish, slightly atrophic macules of the orolabial folds	Melan-A, SOX10, MITF + S100, PRAME−	Discoid LE	Melanocytic pseudonests
Panse et al., 2021 [8]	48/F	Melasma, post-inflammatory hyperpigmentation of the cheek	SOX10, MITF + −	Lichenoid dermatitis	Pseudomelanocytic nests
Panse et al., 2021 [8]	49/M	Erythema multiforme on the cheek	MITF + −	FDE	Pseudomelanocytic nests
Panse et al., 2021 [8]	76/F	Changing lesion on the arm	MITF + −	LP like keratosis	Pseudomelanocytic nests
Panse et al., 2021 [8]	42/M	Rash on the temple	MITF + −	LPP	Pseudomelanocytic nests
Panse et al., 2021 [8]	49/M	Lichen Planus Pigmentosus on the preauricular region	MITF + S100−	LPP	Pseudomelanocytic nests
Panse et al., 2021 [8]	48/M	Irritated SK on the neck	MITF + −	LP like keratosis	Pseudomelanocytic nests
Panse et al., 2021 [8]	56/M	Sarcoidosis of the eyelid	MITF, SOX10 + −	LE	Pseudomelanocytic nests
Panse et al., 2021 [8]	69/F	Lichen planus-like keratosis on the chest	MITF + −	LP like keratosis	Pseudomelanocytic nests
Panse et al., 2021 [8]	69/M	Eczema on the back	MITF, SOX10 + S100−	Graft versus host disease	Pseudomelanocytic nests
Panse et al., 2021 [8]	71/M	Recurrent BCC on the chest	MITF + −	Benign keratosis with reactive changes	Pseudomelanocytic nests
Bertlich et al., 2022 [9]	39/M	Multiple brown macules in a retiform distribution on the left forehead and the left side of the nose and chin	Melan-A, SOX10, MITF, S100, CD68 + TYR, PRAME−	Cutaneus LE	Melanocytic pseudonests
Our case	32/F	Grey-brown patches and macules located on the trunk, axillary flexures, and upper and lower extremities	Melan-A, HMB45, S100, SOX10 + CD68, CD3−	LPP	Melanocytic nests

LE = lupus erythematosus; LPP = Lichen Planus Pigmentosus; FDE = fixed drug eruption; LP = lichen planus.

## Data Availability

No additional data relating to this study is available.
